# Detecting and Evaluating Fatigue Damage Mechanisms in Concrete with Embedded Aggregate Sensors

**DOI:** 10.3390/ma19061201

**Published:** 2026-03-18

**Authors:** Ziwei Song, Shoushan Cheng, Haifang He, Wanheng Li, Yusheng Liu

**Affiliations:** 1 Research Institute of Highway Ministry of Transport, Beijing 100088, China; zw.song@rioh.cn (Z.S.); wh.li@rioh.cn (W.L.); 2 School of Civil Engineering, Beijing Jiaotong University, Beijing 100044, China; 25110398@bjtu.edu.cn

**Keywords:** experimental investigation, compressive fatigue performance, crack location, failure mechanism, fatigue damage

## Abstract

Fatigue damage is a critical factor for the long-term service performance degradation of concrete structures. Nevertheless, the mesoscopic fatigue process is still debatable due to material heterogeneity and the complex internal damage progression. To further investigate the internal damage mechanism of concrete under fatigue loading, this study quantitatively monitors the dynamic internal strain evolution of concrete prismatic specimens during uniaxial compression high-cycle fatigue by designing and embedding aggregate sensors (EAS). The results indicated that EAS may effectively reflect concrete cracking, and the approach can properly capture the internal strain field redistribution features of concrete. Significant internal strain localization was observed during fatigue damage. The turning points in strain evolution, which correlate with the stages of stable propagation and microcrack initiation, were identified. Furthermore, the evolution of internal strain effectively characterized the alteration of stress transfer routes induced by crack propagation. Based on failure modes and mechanical analysis, the synergistic driving mechanism of fatigue damage involving crack growth, interfacial friction and stress field evolution was investigated. The difference in concrete damage under fatigue and monotonic loading due to changing mesoscopic crack propagation was defined, establishing a mechanical foundation for exploring concrete fatigue damage processes. The EAS monitoring method used in this study not only gives a viable approach for the fatigue damage analysis of concrete structures, but it also offers a new viewpoint and data support for comprehending the mesoscopic fatigue mechanism of concrete.

## 1. Introduction

The long-term service stability of concrete structures is strongly influenced by fatigue effects. Fatigue loads have been shown to significantly increase the development and propagation of cracks in key stress zones and the corrosion of reinforcement in medium- and small-span bridges [1,2,3], thereby posing a serious threat to structural durability and safety. Considering the full lifecycle of infrastructure, fatigue damage exhibits irreversible and progressive accumulation, presenting a significant durability concern [4,5]. This detrimental effect is directly observed at the component scale, where the stiffness and load-bearing capacity of concrete progressively degrade with an increasing number of loading cycles [6,7]. Such degradation can lead to premature and brittle failure of materials solely from fatigue damage, without the applied stress ever exceeding the design allowable range [8,9,10,11]. Therefore, an in-depth revelation of the fatigue damage mechanism of concrete materials is a critical scientific prerequisite for improving the long-term reliability of infrastructure.

Concrete fatigue is a complex mechanical process. The *S*-*N* (stress-number) curve is typically used to quantify its core characterization indicators [12,13,14,15]. Accordingly, the evolution of concrete mechanical parameters under cyclic loading, such as damage accumulation, strain development, and stiffness degradation, accounts for a significant portion of the research on fatigue performance [8,16,17,18]. The concrete fatigue responses show a greater dispersion and randomness than the outcomes of static loading tests, according to several macroscopic tests [19,20,21]. Driven by this uncertainty, the research focus has been shifted from descriptive macroscopic phenomena to an in-depth investigation of the intrinsic meso- and micro-scale damage mechanisms.

With the advancement of non-destructive testing and micro-characterization techniques, advanced approaches such as acoustic emission (AE) [22,23,24,25], industrial computed tomography (ICT) [26,27,28,29,30], and scanning electron microscope (SEM) [31,32,33,34] have been widely used to capture the initiation and propagation of internal fatigue damage in concrete. Specifically, Liu et al. [22] utilized AE to analyze the fracture properties and post-peak softening process of rubber concrete under cyclic loading, identifying AE signal characteristics corresponding to internal microcrack propagation. Skarżyński et al. [28] tracked fracture evolution in concrete under compressive fatigue via X-ray micro-CT images, visualizing the spatial and temporal development of internal cracks. Li et al. [31] analyzed the dynamic microstructure deterioration of pavement concrete under fatigue load via SEM, linking micro-crack initiation to macroscopic strength loss. It is widely acknowledged that concrete fatigue failure is essentially a continuous evolutionary process from mesoscopic damage to macroscopic failure. The mechanism lies in the initiation, stable propagation, and eventual coalescence of microcracks at locations such as the interfacial transition zone, leading to the formation of macroscopic cracks [35,36,37], with Zhang [35] establishing the relationship between pore structure and bending fatigue mechanical properties of ordinary concrete, and Qiu et al. [36] conducting a micromechanics-based investigation of engineered cementitious composite fatigue deterioration

Since concrete is a heterogeneous composite material, the effect of material heterogeneity can be explained by the stiffness mismatch between particles and the cement matrix. This difference generates stress concentration in the matrix and the development of local tensile and compressive stresses [18]. Numerous important mechanisms have been identified by research on high-strength concrete. Oneschkow and Timmermann [38] systematically investigated the influence of high-strength concrete and mortar composition on compressive fatigue behavior, confirming that cement matrix damage regulates strain increase during the second stage of fatigue degradation, and the interaction between aggregates and the cement matrix dominates stiffness evolution. The mesoscopic physical mechanism of its fatigue degradation is currently the subject of important debates within the academic community. Inferences based on phenomenological models point out that the evolution and coalescence of microcracks are the precursors to macroscopic fatigue failure. However, there is still no unified understanding of when microcracks start to form systematically. It remains unclear whether they occur at the initial stage of fatigue, as Schäfer and Breitenbücher [39] proposed that high-performance concrete generates microcracks at the initial cyclic loading stage. Similarly, no consensus has been reached about the evolutionary pattern of damage, namely, whether isolated tensile cracks or diffuse compressive damage predominate, with Oneschkow et al. [40] further finding that the dominant damage pattern varies with stress level and concrete composition. These disputes have their origins in part in the shortcomings of conventional experimental techniques. Such approaches cannot accomplish in situ, continuous, and quantitative monitoring of the genuine internal stress–strain state of materials during fatigue loading. Therefore, it is impossible to directly establish the dynamic link between microstructural change and internal mechanical reactions.

This study starts from the internal damage of concrete and regards it as a multiphase composite material to further explore the compressive fatigue damage mechanism of concrete. Prismatic specimens were prepared and subjected to uniaxial compression fatigue tests under various high-cycle fatigue loading conditions. The scientific breakthrough of this work is to track the dynamic and complex internal stress state of concrete by collecting key data such as fatigue life, deformation characteristics and failure modes. Aggregate sensors bonded with strain gauges were used to replace part of the natural aggregates, so as to realize real-time and in situ capture of dynamic strain responses at key internal positions of concrete during the fatigue process. For the internal fatigue damage and force transmission mechanism of concrete, this study explores the relationship between internal strain evolution and microcrack propagation under cyclic loading and reveals the fatigue damage mechanism of concrete from the perspective of internal damage. In addition, this work provides a new experimental perspective for solving the existing disputes on the mesoscopic mechanism of concrete fatigue and highlights the important scientific value of in situ internal monitoring in understanding the essential fatigue behavior of concrete.

## 2. Experimental Program

### 2.1. Materials and Mix Proportions

The designed strength of the test concrete is 40 MPa. The cement used in this study is ordinary Portland cement 42.5R. The coarse aggregate (CA) consists of granite with a particle size of 5 mm to 20 mm, and the fine aggregate (FA) has a particle size range of 0.075 mm to 4.75 mm. The concrete for the tests is self-prepared in the laboratory. The polycarboxylate ether (PCE)-based superplasticizer was used. It exhibits a high water reduction rate and excellent workability retention. The water reduction rate exceeds 25%, and it shows good compatibility with cementitious materials. The mix proportions in this study are shown in Table 1.

### 2.2. Sample Preparation

The fatigue test results exhibit significant dispersion; therefore, it is recommended to use a larger sample size [19] and adopt appropriate statistical methods for result analysis [41,42]. A total of 9 groups of uniaxial compression specimens are designed in this test, as shown in Table 2. Considering that the coefficient of variation of concrete fatigue test data is generally 5–15%, it is recommended to set 5 specimens per group [43]. To ensure the reliability of the test, six specimens are ultimately adopted in each group, including three specimens embedded with aggregate sensors and three cut specimens. In each group, the six specimens are numbered from I to VI, respectively. Specimens I–III belong to Group PA, and Specimens IV–VI belong to Group PQ. The cut specimens are mainly used to observe the crack initiation location and propagation path, so as to verify that the aggregate sensors have no influence on the failure mode of concrete.

The prismatic compression specimens have dimensions of 150 mm × 150 mm × 300 mm. After typical curing for 48 h, the specimens are demolded and then cured for a further 28 days. Six 150 mm cubic specimens are also cast for strength testing; three of them are tested prior to the fatigue tests, and three following them.

**Figure 1:**
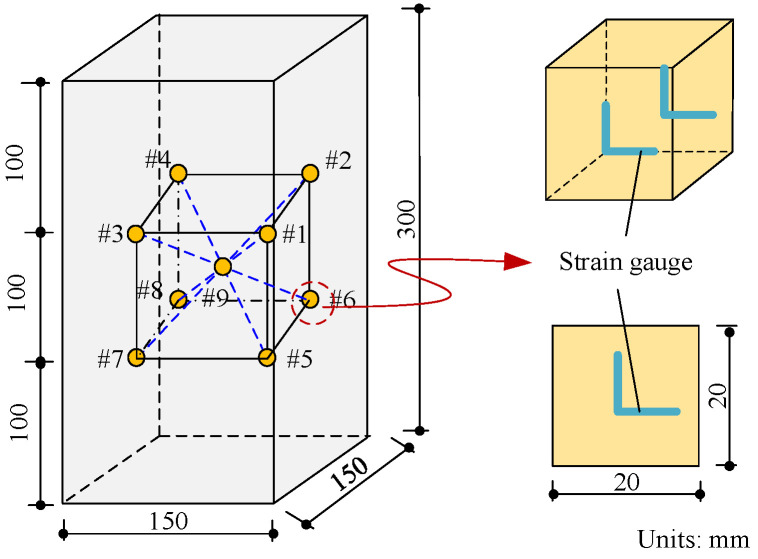
Aggregate sensor strain gauge paste mode and internal aggregate layout.

Nine EAS are embedded into each specimen in accordance with the coarse aggregate framework in order to compare the internal strain variations in concrete under fatigue and monotonic loading. #1 to #9 are the numbers of the aggregates. Four strain gauges are positioned on each of the sensors, which are constructed from 20 mm granite cubes bonded with strain gauges; the precise arrangement is depicted in Figure 1.

**Figure 2:**
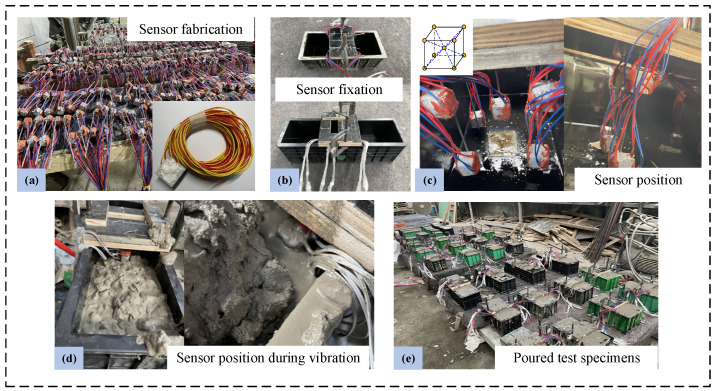
Preparation process of the test specimens: (**a**) Fabrication of aggregate sensors; (**b**) production of the fixed plate; (**c**) positioning of the aggregate sensors; (**d**) position of the aggregate sensor during vibration; (**e**) partially poured test specimens.

Before concrete is poured, the internal aggregate sensors’ locations are calibrated and modified throughout manufacturing. The location and orientation of the sensors are maintained during vibration thanks to the use of thin iron wires that are secured with an air nail gun after passing through the upper wooden plate. The fabrication of aggregate sensors and the concrete casting process are shown in Figure 2.

### 2.3. Test Method

**Figure 3:**
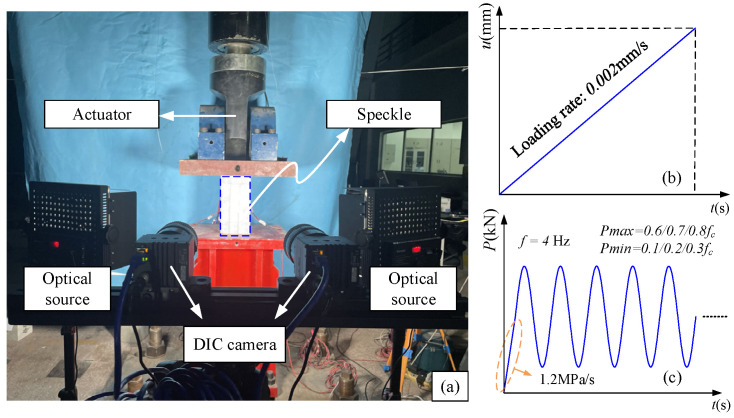
Loading device of test: (**a**) Compression fatigue testing apparatus; (**b**) Static loading; (**c**) Fatigue loading.

As shown in Figure 3, the test parameters chosen to examine the fatigue performance of concrete are the stress amplitude and mean stress level. The fatigue strength of concrete exposed to two million load cycles is roughly 0.6 to 0.65 times its ultimate compressive strength, according to current *S*-*N* curves of concrete fatigue. The maximal stress levels used in this study are 0.6, 0.7, and 0.8 times the compressive strength, respectively, in order to efficiently notice fatigue damage and examine its process in the tests. Meanwhile, to explore the effect of mean stress under the same stress amplitude, the minimum stress levels are set to 0.1, 0.2, and 0.3 times the strength, respectively. The compressive fatigue tests are carried out using a Servotest 100 t electro-hydraulic servo fatigue testing system (Servotest Testing Systems Ltd., Surrey, UK), with a loading frequency of 2–8 Hz. And this testing system is used to apply loads and output load and displacement data.

To acquire the full stress–strain curve for specimens subjected to static loading, an external load is applied in displacement control mode. With the compressive loading rate of 0.002 mm/s, the loading process is kept consistent and continuous. The whole stress–strain curve during static compression is recorded, with the maximum deformation set at 4.0 mm. When the post-peak load dropped to 20% of the peak load, the test was finished.

For fatigue loading, the stress-controlled loading mode is used to better conform to engineering practice. The average compressive strength of the same batch of concrete is used to calculate the stress amplitude. The specimen underwent sinusoidal cyclic loading between *P*_max_ and *P*_min_ after being static loaded to the mean stress level (*P*_max_ + *P*_min_)/2 at a rate of 1.2 MPa/s. The minimal load is set at 0.1*f_c_* in order to reduce zero drift and impact effects. The constant loading frequency of 4 Hz is used.

The number of cycles at which the specimens failed or surpassed 2 million cycles is tracked using a cycle counter during the experiments. The dynamic data collection system tracks all signals synchronously in real time, including load and displacement from the Servotest 100 t testing system, and strain data from external concrete strain gauges and embedded aggregate sensors.

## 3. Result and Discussion

### 3.1. Fatigue Failure Observations

**Figure 4:**
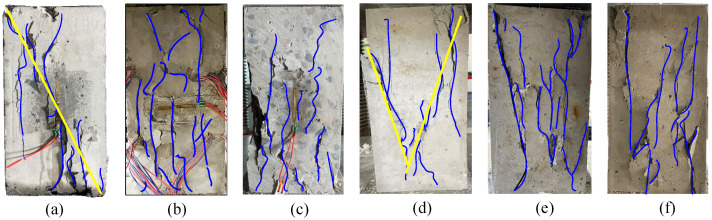
Crack distribution of test specimens before failure: (**a**) Static loading; (**b**) *S*_max_ = 0.6*f_c_*, *S*_min_ = 0.1*f_c_*; (**c**) *S*_max_ = 0.7*f_c_*, *S*_min_ = 0.1*f_c_*; (**d**) *S*_max_ = 0.8*f_c_*, *S*_min_ = 0.1*f_c_*; (**e**) *S*_max_ = 0.7*f_c_*, *S*_min_ = 0.2*f_c_*; (**f**) *S*_max_ = 0.8*f_c_*, *S*_min_ = 0.3*f_c_*.

**Figure 5:**
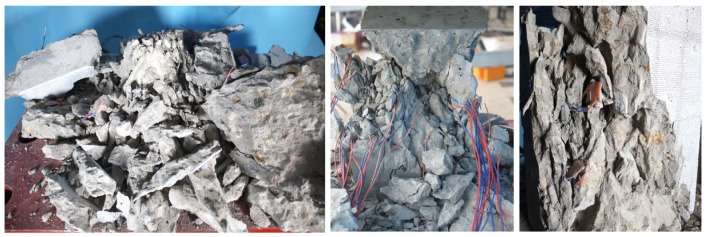
Failure mode of test specimens.

As seen in Figure 4a, the prismatic concrete specimens subjected to uniaxial compression and monotonic stress exhibit shear failure characterized by inclined cracks, but the specimens remain intact overall. As shown in Figure 4b,c, when the load amplitude is relatively low, the compressed fatigue specimens are dominated by vertical splitting failure parallel to the loading direction prior to failure. The specimens are divided into several parallel short columns by vertical splitting cracks, some of which penetrate the aggregate, while most propagate along the interfacial transition zone between aggregate and mortar. With increasing load amplitude, shear or wedge-shaped failure may occur, as presented in Figure 4d. However, when the load amplitude remains unchanged but the mean load level increases, the failure mode of the specimens is basically unchanged. The specimens are still divided into several parallel short columns by vertical splitting cracks, with some cracks penetrating the aggregate and most propagating along the interfacial transition zone between aggregate and mortar, as illustrated in Figure 4b,e,f. As seen in Figure 5, the failure surface has frictional slip lines and the mortar powder that results.

Additionally, specimens subjected to fatigue loading exhibit numerous microcracks. In contrast, macrocracks are more evenly and dispersedly distributed, with the dominant diagonal cracks that develop during monotonic compression. This could be explained by the persistently reduced stress level, which causes numerous interior microcracks to begin and spread. Under cyclic pressure, these microcracks repeatedly open and close, and randomly dispersed secondary cracks progressively combine to create larger cracks that finally connect and penetrate, causing specimen failure. A more complex failure pattern results from the specimen’s tendency to fail in a splitting mode when the stress amplitude is lower. The failure mode approaches that occur under monotonic loading as the stress amplitude increases. The failure mode of fatigue specimens, which all show cracks parallel to the loading direction, is not significantly affected by changing the mean stress level under the same stress amplitude.

### 3.2. Fatigue Life

**Figure 6:**
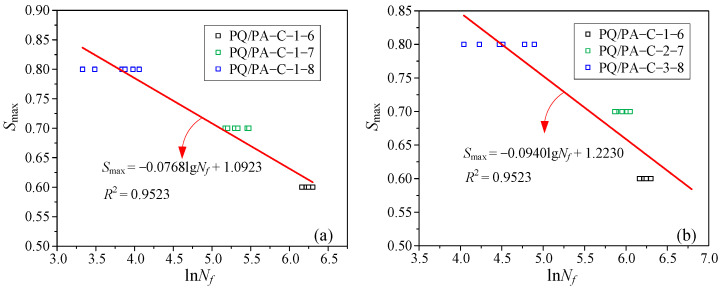
*S*-*N* curves of compression test specimens: (**a**) Test group ②③④; (**b**) test group ②⑤⑤.

The test results indicated that the fatigue life of concrete is affected by both stress amplitude and mean stress level, which is different from the fatigue behavior of steel bars. Computational mistakes will result from establishing an *S*-*N* curve based only on the maximum stress. As a result, as illustrated in Figure 6, the *S*-*N* curve, which is defined by the stress ratio *R* as well as the maximum stress level *S*_max_, is used in this work to fit the data. The fitted parameters agree with the commonly recognized range (0.064–0.080) described in Reference [44]. This outcome further demonstrates that the integrated aggregate sensors have no appreciable impact on concrete fatigue performance.

(1) $$S_{\max}=1-0.0701\left(1-R\right)\mathrm{lg}N$$

### 3.3. Failure Deformation

#### 3.3.1. Stress–Strain Relation of Sample

**Figure 7:**
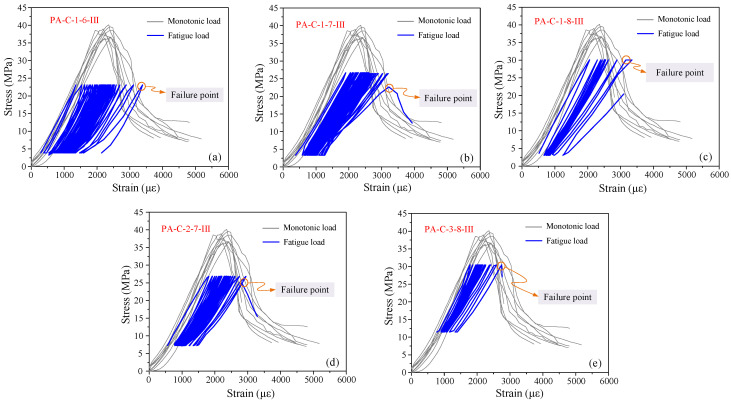
Superposed monotonic and fatigue stress–strain curves: (**a**) PA-C-1-6; (**b**) PA-C-1-7; (**c**) PA-C-1-8; (**d**) PA-C-2-7; (**e**) PA-C-3-8.

The stress–strain curves are plotted against the monotonic loading curve, as seen in Figure 7, using one specimen from each group whose fatigue life is near the average value. The monotonic curve and the fatigue curve essentially match at the beginning. The curves of fatigue loading progressively move to the right as the cycle number increases, accompanied by ongoing strain accumulation. The strain per cycle rises, the curve’s slope falls, the unloading stiffness sharply declines, and the curve becomes sparser as fatigue failure approaches. Eventually, a sudden rise in tension causes the specimen to fail.

According to the results in References [45,46,47], fatigue specimens under various stress conditions all have peak strains at failure that are comparable to those in the descending branch under monotonic loading. This suggests that the maximum stress level is the primary factor influencing the fatigue failure strain of concrete under compression. Consequently, it is reasonable to conclude that the strain on the descending branch of the monotonic stress–strain curve at the appropriate peak stress level corresponds to the peak strain of concrete at failure under fatigue loading. Thus, the strain values in the descending branch can serve as a guide for figuring out the concrete’s fatigue failure strain.

#### 3.3.2. Evolution Curve of Fatigue Strain

**Figure 8:**
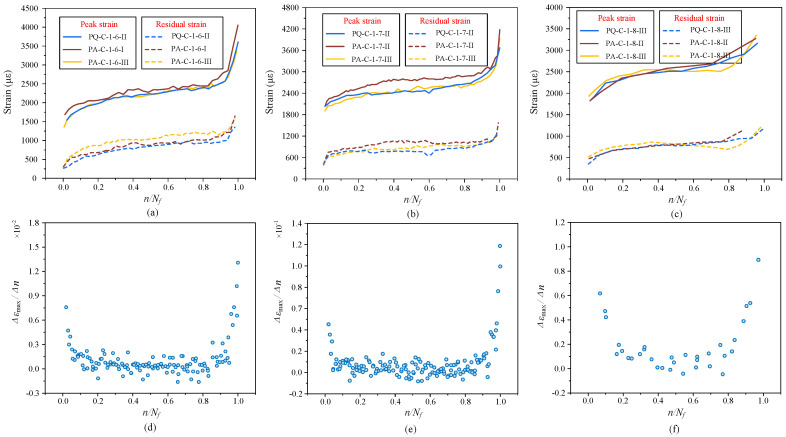
The evolution of fatigue strain and strain rate under different constant stress amplitudes: (**a**) strain evolution curve of *P*_min_ = 0.1*f_u_*, *P*_max_ = 0.6*f_u_*; (**b**) strain evolution curve of *P*_min_ = 0.1*f_u_*, *P*_max_ = 0.7*f_u_*; (**c**) strain evolution curve of *P*_min_ = 0.1*f_u_*, *P*_max_ = 0.8*f_u_*; (**d**) strain evolution rate curve of *P*_min_ = 0.1*f_u_*, *P*_max_ = 0.6*f_u_*; (**e**) strain evolution rate curve of *P*_min_ = 0.1*f_u_*, *P*_max_ = 0.7*f_u_*; (**f**) strain evolution rate curve of *P*_min_ = 0.1*f_u_*, *P*_max_ = 0.8*f_u_*.

A clear and useful indicator of concrete fatigue behavior is strain. The uniaxial strain and its evolution rate during the course of uniaxial compression fatigue are shown in Figure 8a–c. Both the peak strain and residual strain increase with the number of cycles at various maximum stress levels, following a standard three-stage development rule. This further confirms the validity of the current tests by being in line with previous research findings.

It is noteworthy that the strain values of specimens before failure (at the conclusion of the second stage) do not noticeably alter when the maximum stress level rises from 0.6*f_c_* to 0.7*f_c_* and 0.8*f_c_*. With a high degree of uniformity, the residual strain varies from 800 με to 1300 με, while the peak strain concentrates in the range of 2500 με to 2800 με. This suggests that strain may function as a more stable and trustworthy critical index for fatigue failure and can be used to assess the fatigue damage state, in contrast to fatigue life, which shows significant scatter.

As seen in Figure 8e,f, the strain evolution rate is rather high during the initial loading stage, rapidly drops thereafter, and then rises significantly close to failure. The strain rate in this test still shows nonlinear fluctuation in this stage, in contrast to the conclusion of a constant evolution rate in the second stage stated in other investigations. It rapidly rises after a sluggish decline throughout the first half of the fatigue life. This pattern offers a fresh foundation for assessing the fatigue damage process. The strain rate transition from declining to increasing can be seen as a sign that the fatigue life has passed the halfway point, which is helpful for determining how much fatigue life is left.

**Figure 9:**
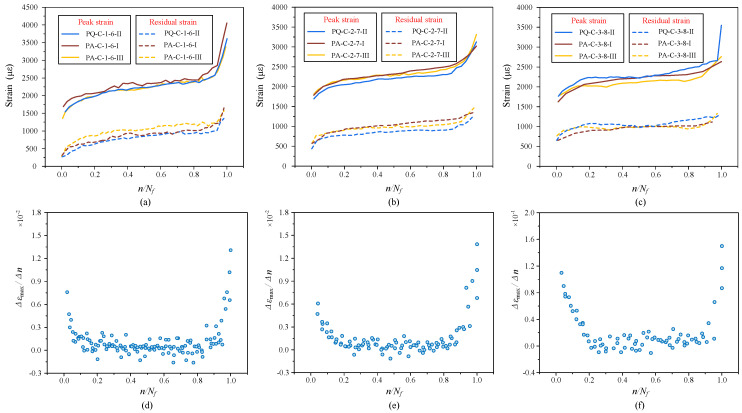
The evolution of constant amplitude fatigue strain under different mean stress levels (with identical stress amplitude): (**a**) strain evolution curve of *P*_min_ = 0.1*f_u_*, *P*_max_ = 0.6*f_u_*; (**b**) strain evolution curve of *P*_min_ = 0.2*f_u_*, *P*_max_ = 0.7*f_u_*; (**c**) strain evolution curve of *P*_min_ = 0.3*f_u_*, *P*_max_ = 0.8*f_u_*; (**d**) strain evolution rate curve of *P*_min_ = 0.1*f_u_*, *P*_max_ = 0.6*f_u_*; (**e**) strain evolution rate curve of *P*_min_ = 0.2*f_u_*, *P*_max_ = 0.7*f_u_*; (**f**) strain evolution rate curve of *P*_min_ = 0.3*f_u_*, *P*_max_ = 0.8*f_u_*.

For the same stress amplitude, raising the mean stress level results in a minor increase in both peak and residual strains while maintaining the three-stage evolution pattern, as shown in Figure 9a–c. Theoretically, greater plastic strain should arise from a higher mean stress since it indicates that the stress level is closer to the ultimate strength. The little increase in peak strain that has been seen, however, might be connected to the variation in fatigue life. For instance, Groups ②, ⑤, and ⑥ have average fatigue lifetimes of 1,751,769, 918,810, and 37,968 cycles, respectively. Elastic-plastic strain and strain accumulated with prolonged loading make up the total peak strain, which is dependent on the number of cycles, constitutive behavior, damage evolution, and stress intensity.

Although the strain rate rises with increasing mean stress under the same stress amplitude, it is generally much lower than that of the test groups in Figure 8, according to the strain evolution rate perspective, as shown in Figure 9. The second-stage strain rates are only 6.3 × 10^−4^ and 5.9 × 10^−4^, respectively, even when the maximum stress approaches 0.7*f_c_* and 0.8*f_c_*. This could be explained by the fact that the total strain evolution rate is suppressed when the stress amplitude is fixed. The stress variation per cycle is limited, resulting in more uniformly distributed damage inside the material and weaker localized crack growth. Additionally, under cyclic loading, concrete may display a certain adaptation or “hardening” effect that stabilizes the material response and further lowers the strain rate fluctuation magnitude.

#### 3.3.3. Evolution Curve of Elastic Modulus and Damage

The modulus of deformation for each cycle is calculated using the following equation,

(2) $$E_{i}=\frac{\sigma_{\max}-\sigma_{\min}}{\varepsilon_{\max}^{i}-\varepsilon_{\min}^{i}}=\frac{\Delta \sigma }{\Delta \varepsilon_{i}}$$

where Δσ is the stress amplitude and $\Delta \varepsilon_{i}$ is the strain amplitude at the *i*-th cycle. The deformation modulus *E_i_* is negatively correlated with the strain variation $\Delta \varepsilon_{i}$. As fatigue loading proceeds, the strain amplitude increases gradually, and *E_i_* decreases with the increase in loading cycles.

To quantify the damage evolution of concrete under cyclic loading, the fatigue damage variable *D_f_* is defined as the relative stiffness degradation of concrete,

(3) $$D_{f}=1-\frac{E_{i}}{E_{0}}$$

where *D_f_* is the fatigue damage variable; *E_i_* is the secant modulus of the unloading curve at the *i*-th cycle; *E*_0_ is the initial secant modulus, defined as the secant modulus of the first unloading cycle.

**Figure 10:**
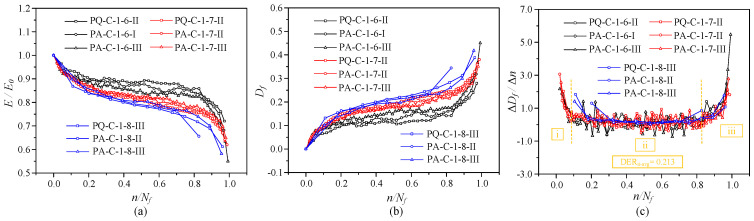
The degradation of elastic modulus and the accumulation of damage under fatigue loading with different stress amplitudes: (**a**) Elastic modulus degradation; (**b**) damage evolution; (**c**) damage evolution rate.

Figure 10 shows the normalized relationships between cycle ratio, fatigue damage, and elastic modulus. Because fatigue microcracks propagate under varying stress amplitudes, the normalized secant modulus falls with increased loading cycles. The final residual modulus is roughly 55–75% of the initial elastic modulus *E*_0_. In the first and second phases, modulus deterioration is accelerated by a larger stress amplitude. In the third stage, it is slowed down. The elastic modulus gradually deteriorates at high stress levels because damage builds up relatively uniformly and slowly. A higher stress level results in a bigger residual strain at the same cycle ratio *n*/*N_f_*, which further lowers the residual modulus. The damage evolution shows a three-stage growth trend: the three-stage characteristics become less evident, and the damage rate increases with increasing stress amplitude. It should be mentioned that in the last cycle, specimens fail suddenly, and the damage value falls short of unity.

**Figure 11:**
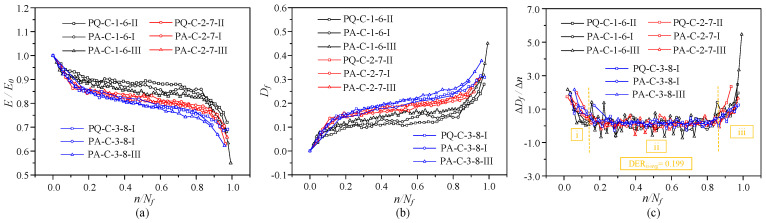
The degradation of elastic modulus and the accumulation of damage under fatigue loading with different mean stress levels (with identical stress amplitude): (**a**) Elastic modulus degradation; (**b**) damage evolution; (**c**) damage evolution rate.

The normalized associations between damage evolution and modulus degradation for specimens with varied mean stress levels and constant stress amplitude are shown in Figure 11. The large stress level causes the elastic modulus to decline more quickly and the damage to evolve more rapidly for the same stress amplitude. Concrete internal damage occurs more slowly and at a slower modulus decay rate when the mean stress is reduced, leading to more stable mechanical performance.

### 3.4. Fatigue Damage Mechanism

Concrete macroscopic mechanical properties are described by conventional mechanical tests. These tests do not account for the internal mechanical behavior. Concrete degradation occurs at the mesoscale when internal cracks continue to spread and develop, changing the concrete’s local deformation properties. Complex stress transfer between individual aggregate particles is induced by external loading. Consequently, the internal damage and stress condition of concrete can be implicitly described using the strain (stress) evolution of EAS.

#### 3.4.1. Redistribution of Internal Stress

At the mesoscale, the stress distribution between aggregate and mortar can be described by composite material models,

(4) $$\sigma_{a}=\frac{E_{a}}{E_{eff}}\sigma ,\sigma_{m}=\frac{E_{m}}{E_{eff}}\sigma$$

where *E_a_* and *E_m_* denote the elastic moduli of aggregate and mortar, respectively. *E_eff_* represents the effective modulus of the composite material. Damage-induced degradation of *E_eff_* gives rise to an increase in the stress partition ratio $\sigma_{a}/\sigma$ of the aggregate phase.

Local stiffness drops and stress is shifted toward the aggregate phase as microcracks start and spread at the interfacial transition zone or inside the mortar matrix. Strong non-uniformity is seen in the stress partitioning between aggregate and cement paste, which no longer follows the ratio of their respective elastic moduli. As shown in Equation (5), the incremental additional strain Δ*ε_a_* is related to the damage variable increment Δ*D* based on continuum damage mechanics (CDM) [48]. This derivation follows the strain equivalence principle, where the effective elastic modulus *E_eff_* decreases with the accumulation of damage *D*.

(5) $$\Delta \varepsilon_{a}=\frac{\Delta \sigma_{a}}{E_{a}}=\frac{1}{E_{a}}\left(\frac{\Delta E_{eff}^{-1}}{\partial E_{eff}/\partial D}\right)\Delta D\cdot \sigma$$

An increase in strain difference indicates that the local damage increment is spatially non-uniformly distributed.

(1) Static Loading

**Figure 12:**
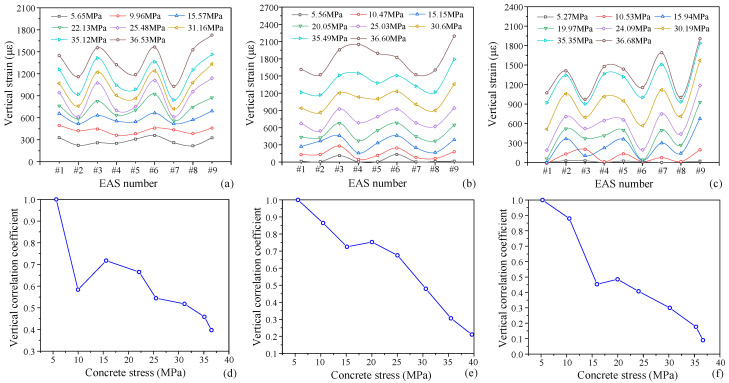
The distribution curve of vertical strain in the aggregate of test specimens: (**a**) P-S-7; (**b**) P-S-8; (**c**) P-S-9; and the variation in correlation coefficients under monotonic loading: (**d**) P-S-7; (**e**) P-S-8; (**f**) P-S-9.

Only the strain data from aggregate sensors during the ascending branch of the concrete stress–strain curve under monotonic loading are analyzed to study the internal damage and stress transfer mechanism of concrete. Figure 12 illustrates the vertical stresses observed by each aggregate sensor during the monotonic compression test on concrete prisms.

The vertical stresses of aggregate at various locations inside concrete during monotonic compression show discernible variations, indicating the variance in load transmission paths and directly influencing the form of cracking and failure. The stresses of various aggregate particles are comparatively close at low load levels (stress < 0.4*f_c_*). The strain rate dramatically increases as the stress approaches the peak value, suggesting that as concrete reaches the plastic stage, internal damage occurs and microcracks merge, leading to stress redistribution and an increased strain difference among aggregate particles.

The aggregate particles with larger strains (No. #1, #3, #5, #8, #9; #3, #4, #5, #6, #9 and #2, #4, #5, #7, #9, respectively) in specimens P-S-7, P-S-8, and P-S-9 are dispersed along a diagonal pattern, which is compatible with the normal uniaxial compression failure mode. This suggests that interior damage can be implicitly described by aggregate strain. Among them, aggregate #9 exhibits the most strain. This is because it serves as a crucial component and is situated in the middle of the load-transfer line. There is only one aggregate in this layer to support the weight, which causes a large concentration of stress.

The difference between the aggregate strain distribution at different loading cycles and the starting distribution was analyzed using the correlation coefficient in order to measure the stress redistribution caused by cracking, as shown in Figure 12b,d,f. A steadily growing gap in the stress distribution is indicated by the continuous decline in the correlation coefficient. At first, damage is homogeneous, but it eventually localizes along the primary crack propagation channel, creating strain concentration zones. Concrete damage under monotonic loading is controlled by localized large cracks that initiate, evolve, and eventually fail, as seen by the monotonic reduction in the correlation coefficient.

(6) $$r\left(\varepsilon \right)=\frac{\mathrm{cov}\left(\varepsilon_{i},\varepsilon_{0}\right)}{\sqrt{\mathrm{var}\left(\varepsilon_{i}\right)\mathrm{var}\left(\varepsilon_{0}\right)}}$$

where $\varepsilon_{i}$ is the peak strain (transverse and vertical) of each aggregate under subsequent load levels, $\varepsilon_{0}$ is the peak strain (transverse and vertical) of each aggregate in the elastic stage.

**Figure 13:**
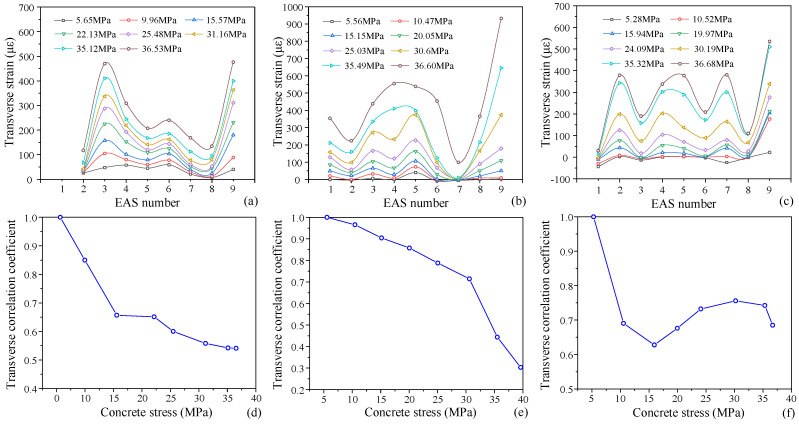
The distribution curve of transverse strain in the aggregate of test specimens: (**a**) P-S-7; (**b**) P-S-8; (**c**) P-S-9; and the variation in correlation coefficients under monotonic loading: (**d**) P-S-7; (**e**) P-S-8; (**f**) P-S-9.

Similarly, Figure 13 displays the transverse strain distribution curves of aggregates. Although the transverse strain of concrete under uniaxial compression is generally quite minor, its fluctuation trend is comparable to that of the vertical strain. Larger strain aggregate particles are #3, #5, #8, #9 in specimen P-S-7. #3, #4, #5, #6, #9 in P-S-8; and #2, #4, #5, #7, #9 in P-S-9 (data for P-S-3 are missing due to strain gauge failure). The lateral constraint on the aggregate is weakened by the cracking because microcracks under uniaxial compression are roughly parallel to the loading direction. This causes the transverse strain to vary more than the vertical strain, which makes the transverse strain a better indicator of the distribution of internal cracks.

The aggregate strain correlation coefficient exhibits a generally declining trend, suggesting a progressive expansion of the stress distribution disparity. The correlation coefficient for specimen P-S-9 first falls and then marginally increases. This could be explained by the fact that damage is first focused in one direction; as the load increases, damage progressively spreads to other areas and eventually tends to propagate along the primary crack once more, leading to a recurrent fall in the later stage.

The aggregate transverse strain distribution curves are shown in Figure 13. Concrete transverse strain is comparatively minimal under uniaxial compression, although its variation trend is usually in line with the vertical strain. The following aggregate particles have higher strains: #3, #5, #8, #9 in specimen P-S-7; #3, #4, #5, #6, #9 in specimen P-S-8; and #2, #4, #5, #7, #9 in specimen P-S-9. Data for specimen P-S-3 are missing due to strain gauge failure.

The microcracks in the concrete are approximately parallel to the loading direction when subjected to uniaxial compression loading. The transverse strain of aggregates will increase as a result of the cracks that are created around them. As a result, the transverse strain of aggregate variation range is more important than their vertical strain variation range, and it more accurately depicts the distribution of internal cracks in the concrete.

Overall, there is a downward trend in the correlation coefficient of the transverse strain in aggregate. The correlation coefficient for specimen P-S-9 first declines noticeably before marginally increasing. Damage is initially concentrated in one direction, which explains this. A fresh decrease occurs in the later stage of the curve as a result of damage that progressively spreads to additional areas as the load increases and eventually tends to localize along the primary fracture path.

(2) Fatigue Loading

**Figure 14:**
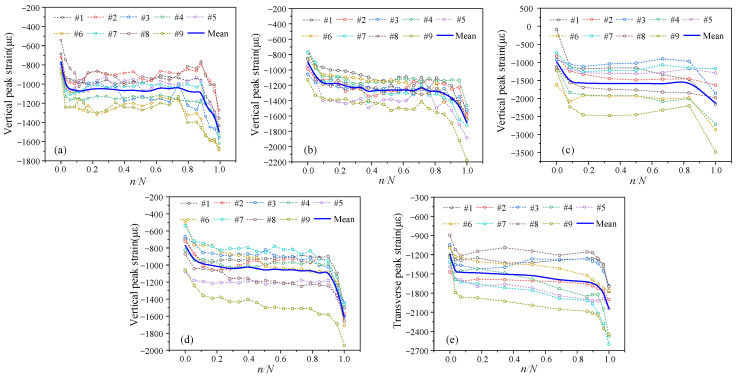
The vertical peak strain of aggregates under constant amplitude cyclic loading: (**a**) PA-C-1-6; (**b**) PA-C-1-7; (**c**) PA-C-1-8; (**d**) PA-C-2-7; (**e**) PA-C-3-8.

**Figure 15:**
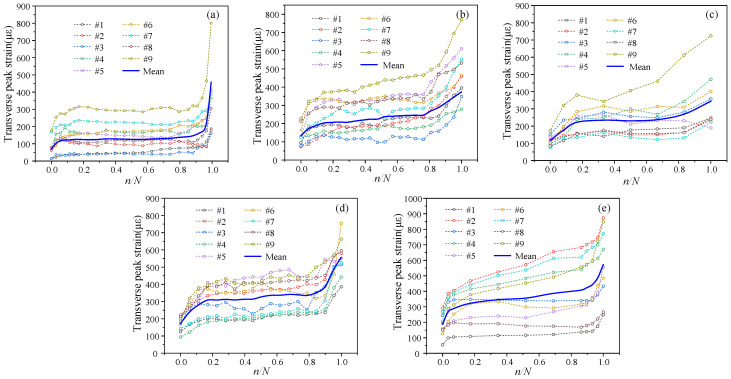
The transverse peak strain of aggregates under constant amplitude cyclic loading: (**a**) PA-C-1-6; (**b**) PA-C-1-7; (**c**) PA-C-1-8; (**d**) PA-C-2-7; (**e**) PA-C-3-8.

**Figure 16:**
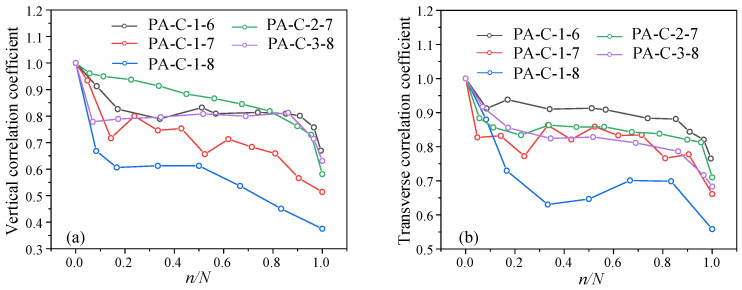
The aggregate correlation coefficients under different amplitude levels: (**a**) Comparison of vertical correlation coefficient; (**b**) comparison of transverse correlation coefficient.

The stress states of the aggregate at various locations during fatigue loading varied even at the same external strain level, as shown in Figure 14, Figure 15 and Figure 16. Three-stage evolution characteristics are seen in the aggregate strain correlation coefficient as well as the vertical and transverse strains of individual aggregate particles. Microcracks are randomly initiated during the initial fatigue stage due to the activation of initial internal micro-defects and interfacial debonding. The correlation coefficient rapidly drops as a result of the uneven strain increase among various aggregate particles, suggesting that significant stress redistribution occurs and the uniform stress state is disturbed. Microcracks enter a steady propagation regime during the intermediate fatigue stage. The aggregate and mortar create a dynamic mesoscale mechanical equilibrium with stable macroscopic strain. As a result, the correlation coefficient gently fluctuates before entering a stable phase. Dispersed microcracks combine to become dominant macrocracks at the late fatigue stage, which causes a sudden shift in the load-transfer pathways and extreme stress concentration in a small number of aggregate particles. A rapid decline in the correlation coefficient indicates that deterioration has reached an unstable stage and will ultimately result in specimen failure.

Figure 16 illustrates how the stress amplitude influences the correlation coefficients for both vertical and transverse strain. The correlation coefficient decreases more significantly with increasing stress amplitude. Increasing mean stress has a negligible effect on the correlation coefficient under the same stress amplitude. Because the strengths of the three components of concrete—aggregate, mortar, and interface—differ, when the stress is greater than the strength of one component, that component is destroyed and can no longer support the weight, which causes the stress to be redistributed to other phases. The stress amplitude mainly influences the fatigue damage of mortar and the interface because the strength and modulus of aggregate are significantly higher than those of mortar and the interfacial transition zone. High stress amplitudes cause damage to worsen and the stress concentration in fractured areas to become more noticeable, as seen in Figure 16a, which accelerates the decline of the correlation coefficient. The correlation coefficient drops more gradually for low stress amplitudes because damage develops gradually, and crack propagation is more uniform.

It is worth noting that although a high stress level accelerates crack initiation, damage evolution becomes more consistent under the same stress amplitude, as shown in Figure 16b. At a given stress amplitude, raising the stress level also reduces the strain correlation coefficient, but the magnitude is significantly smaller than that caused by varying the stress amplitude. This indicates that the influence of stress level on internal damage and strain field evolution is relatively minor.

After *n*/*N* > 0.5, the decreasing rate of the correlation coefficient under high stress becomes greater than that under low stress. The correlation coefficient shows a significantly greater decrease at high stress levels. As a metric for assessing fatigue damage in concrete, the correlation coefficient seems to be more sensitive to high-risk situations, which is in line with realistic engineering needs.

#### 3.4.2. Discussion of the Fatigue Damage Process

**Figure 17:**
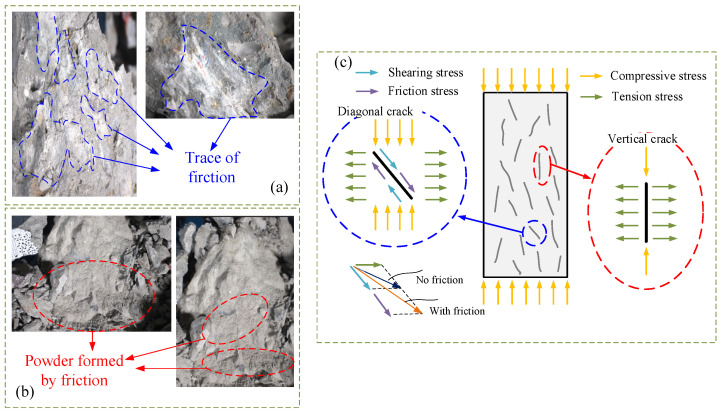
Typical morphology of failure surface of concrete sample subjected to compressive fatigue loading and stress analysis of crack interface: (**a**) Failure surface in this test; (**b**) friction powder in this test; (**c**) stress analysis of crack interface.

There is a noticeable crack surface effect in microcracks under cyclic loading. When rough fracture surfaces close during unloading, frictional forces are created under compression. As seen in Figure 17, repeated cyclic action creates wear debris inside the cracks, which modifies the direction and stress transfer. The non-uniform stress distribution caused by microcracks controls the failure mechanism of concrete.

The frictional behavior of rough surfaces during crack closure can be described by the following model. The normal stress on the crack surface is $\sigma_{n}$. The relative tangential displacement is *δ*, and the frictional energy dissipation rate density is ${\dot{\omega }}_{f}$,

(7) $${\dot{\omega }}_{f}=\mu_{eff}\sigma_{n}\dot{\delta }\cdot H\left(\sigma_{n}\right)$$

where $\mu_{\mathrm{eff}}$ is the effective friction coefficient, $H\left(\sigma_{n}\right)$ is the Heaviside function (friction exists only when $\sigma_{n}$ > 0, i.e., when the crack is under compression). The effective friction coefficient evolves with the number of cycles,

(8) $$\mu_{eff}\left(N\right)=\mu_{0}\exp\left(-\alpha N\right)+\mu_{\infty }\left[1-\exp\left(-\alpha N\right)\right]$$

where $\mu_{0}$ is the initial friction coefficient, $\mu_{\infty }$ is the steady-state friction coefficient, α is the attenuation coefficient.

The wear rate is proportional to the frictional energy dissipation and is also influenced by the roughness of the crack surface.

(9) $$\frac{dV_{\omega }}{dN}=\beta \int_{A}{\dot{\omega }}_{f}dA\cdot R_{a}\left(N\right)$$

where *V_m_* is the volume of wear particles, $\beta_{\omega }$ is the wear coefficient, $R_{a}\left(N\right)$ is the mean surface roughness, which decays with the number of cycles.

(10) $$R_{a}\left(N\right)=R_{a0}\exp\left(-\gamma N\right)+R_{a\infty }$$

The presence of wear particles alters the contact conditions on the crack surfaces, which in turn affects stress transfer.

Wear particles cause random deflection in the direction of stress transfer. Let the initial load transfer direction be $\theta_{0}$, the deflected direction θ follows a normal distribution.

(11) $$f\left(\theta \right)=\frac{1}{\sqrt{2\pi }\sigma_{\theta }}\exp\left[-\frac{{\left(\theta -\theta_{0}\right)}^{2}}{2\sigma_{\theta }^{2}}\right]$$

This randomization of the stress transfer direction is a key reason why the strain distribution correlation coefficient remains relatively stable in the middle stage of fatigue loading, rather than decreasing monotonically.

Static failure and fatigue damage mechanisms are fundamentally different. Static damage tends to localize and develop directly toward a single major crack, while energy dissipation shows spatial concentration and temporal instantaneity under monotonic stress. According to Equation (12), the energy dissipation is mainly caused by the crack formation energy that is released intensively at the moment of failure. By contrast, fatigue damage is diffuse and cumulative, involving repeated crack opening/closure, surface wear, and dynamic mesoscale equilibrium. The total dissipated energy under fatigue loading consists of three components: hysteretic energy due to matrix viscoelasticity and microplasticity, frictional energy dissipation on crack surfaces, and energy consumed by incremental crack growth, as expressed in Equation (13).

(12) $$W_{mono}=\int_{0}^{\varepsilon_{f}}\sigma d\varepsilon =\frac{1}{2}E_{0}\varepsilon_{y}^{2}+\int_{\varepsilon_{y}}^{\varepsilon_{f}}\sigma d\varepsilon +G_{f}/l_{c}$$

where $\varepsilon_{y}$ is the yield strain, *G_f_* is the fracture energy, and *l_c_* is the characteristic length. The dissipated energy under monotonic loading is dominated by crack formation energy, which is released intensively at the instant of failure.

(13) $$W_{fat}=\sum_{i=1}^{N}\oint_{V}{\dot{w}}_{f}dVdN+\sum_{i=1}^{N}\oint_{V}{\dot{w}}_{p}dVdN+\oint_{V}g_{f}dV$$

where *dV* is the volume element, *dN* is the number of cycles, ${\dot{\omega }}_{p}$ is the plastic energy dissipation rate density, and *g_f_* is the surface energy density of newly formed cracks.

In summary, the three-stage evolution of the aggregate strain correlation coefficient is essentially a macroscopic manifestation of the internal stress field in concrete transitioning from uniform distribution, through dynamic equilibrium, to localized concentration under fatigue loading. The three-stage characteristic of the mesoscopic stress field evolution distinguishes it fundamentally from monotonic damage mechanisms. A thorough understanding of this process, particularly the dynamic equilibrium behavior at crack interfaces, is critical for evaluating the fatigue performance of concrete structures.

## 4. Conclusions

This research describes a method that uses aggregate sensors to track the dynamic stress of concrete in reinforced concrete structures under cyclic loading. This study investigates the relationship between internal strain development and microcrack propagation in concrete under repeated loading and explores the fatigue damage mechanism from the perspective of internal damage by using embedded aggregate sensors to record the evolution of the internal strain field. The following are the primary conclusions,

(1) EAS can effectively capture the evolution of internal microcracks and the load transfer mechanism in concrete. Under fatigue loading, the correlation coefficient of the strain from the sensors exhibits a three-stage degradation, providing a feasible approach for fatigue damage monitoring of practical structures.
(2) Static failure under uniaxial compression is characterized by a single diagonal crack. By contrast, fatigue failure initiates with multiple vertical cracks and eventually leads to brittle disintegration. Frictional traces are observed on the fracture surfaces, indicating the occurrence of shear friction.
(3) The critical strain for fatigue failure is defined on the descending branch of the monotonic stress–strain curve at peak load. At failure, the deformation modulus drops to 55–75% for compression specimens and 40–50% for bending specimens, independent of stress amplitude and mean stress. A reversal of the strain evolution rate from decreasing to increasing indicates that the fatigue life has passed the halfway point.
(4) A larger stress amplitude causes more severe degradation of the aggregate strain correlation coefficient, more localized stress, and concentrated crack growth, while a smaller amplitude leads to more uniform damage. Mean stress has little effect at the same stress amplitude.
(5) Concrete fatigue damage is governed by coupled effects of accumulated damage, crack interface friction, and internal stress redistribution. Under compressive fatigue, this coupling results in behavior distinctly different from static failure.
(6) Internal strain evolution effectively reflects changes in stress transfer paths due to crack propagation. Concrete fatigue damage arises from coupled crack accumulation, interface friction, and stress field evolution, providing a mechanical basis for fatigue analysis.
(7) The research on aggregate sensors can be further expanded and deepened from multiple perspectives, facilitating their long-term deployment in monitoring the internal state of concrete members and establishing a quantitative mapping model between sensor signals and structural health conditions.

## Figures and Tables

**Table 1:** Material mix proportions of concrete.

Water (kg/m^3^)	Cement (kg/m^3^)	W/C	FA (kg/m^3^)	CA (kg/m^3^)	SR	SP (kg/m^3^)
187.5	468	0.40	797	859.5	48.1%	6.3

Notes: W/C refers to the water-to-cement ratio, SR and SP denote the sand ratio and superplasticizer, respectively.

**Table 2:** Design parameters of the test specimens.

Load Type	Test Group	Specimens	*S*_max_	*S*_min_	No.
Static loading	①	P-S	-	-	3(Standard) + 3(Cutting) +3(EAS)
Constant amplitude fatigue loading	②	PQ-C-1-6/PA-C-1-6	0.6*f_c_*	0.1*f_c_*	3(Cutting) + 3(EAS)
	③	PQ-C-1-7/PA-C-1-7	0.7*f_c_*	0.1*f_c_*	3(Cutting) + 3(EAS)
	④	PQ C-1-8/PA-C-1-8	0.8*f_c_*	0.1*f_c_*	3(Cutting) + 3(EAS)
	⑤	PQ-C-2-7/PA-C-2-7	0.7*f_c_*	0.2*f_c_*	3(Cutting) + 3(EAS)
	⑥	PQ-C-3-8/PA-C-3-8	0.8*f_c_*	0.3*f_c_*	3(Cutting) + 3(EAS)

Notes: P denotes prismatic compression specimens; Q denotes cut specimens; A denotes specimens with aggregate sensors; C denotes constant-amplitude loading; *f_c_* is the average compressive strength of the nine groups of concrete prisms, which is 38.12 MPa.

## Data Availability

The original contributions presented in this study are included in the article. Further inquiries can be directed to the corresponding authors.

## References

[1] Bazant Z.P., Hubler M.H. (2014). Theory of cyclic creep of concrete based on Paris law for fatigue growth of subcritical microcracks. J. Mech. Phys. Solids.

[2] Chen H., Zhan X., Zhu X., Zhang W. (2022). Fatigue evaluation of steel-concrete composite deck in steel truss bridge—A case study. Front. Struct. Civ. Eng..

[3] Chen H., Sun Z., Zhang X., Fan J. (2023). Tensile Fatigue Properties of Ordinary Plain Concrete and Reinforced Concrete under Flexural Loading. Materials.

[4] Cui C., Xu Y.L., Zhang Q.H., Wang F.Y. (2020). Vehicle-induced fatigue damage prognosis of orthotropic steel decks of cable-stayed bridges. Eng. Struct..

[5] Shi H., Li H., Huang F., Yang Z., Dong H., Wang Z., Wen J., Li L., Yi Z. (2024). Mechanical properties and microstructure evolution of recycled sand concrete under high-frequency flexural fatigue load. J. Build. Eng..

[6] Li L., Hou B., Lu Z., Liu F. (2018). Fatigue behaviour of sea sand concrete beams reinforced with basalt fibre-reinforced polymer bars. Constr. Build. Mater..

[7] Shi J., Cui C., Song L., Liu M., Yu Z. (2025). Static-Fatigue Unified Damage Constitutive Model of Concrete Based on Accumulated Energy Equivalent Strain. J. Eng. Mech..

[8] Banjara N.K., Ramanjaneyulu K. (2018). Experimental Investigations and Numerical Simulations on the Flexural Fatigue Behavior of Plain and Fiber-Reinforced Concrete. J. Mater. Civ. Eng..

[9] Carlesso D.M., Cavalaro S., de la Fuente A. (2021). Flexural fatigue of pre-cracked plastic fibre reinforced concrete: Experimental study and numerical modeling. Cem. Concr. Compos..

[10] Kasu S.R., Deb S., Mitra N., Muppireddy A.R., Kusam S.R. (2019). Influence of aggregate size on flexural fatigue response of concrete. Constr. Build. Mater..

[11] Tavakoli H.R., Mahmoudi S., Goltabar A.R., Jalali P. (2017). Experimental evaluation of the effects of reverse cyclic loading rate on the mechanical behavior of reinforced SCC beams. Constr. Build. Mater..

[12] Zhang B., Phillips D.V., Wu K. (1996). Effects of loading frequency and stress reversal on fatigue life of plain concrete. Mag. Concr. Res..

[13] Saucedo L., Yu R.C., Medeiros A., Zhang X., Ruiz G. (2013). A probabilistic fatigue model based on the initial distribution to consider frequency effect in plain and fiber reinforced concrete. Int. J. Fatigue.

[14] Lee M.K., Barr B.I.G. (2004). An overview of the fatigue behaviour of plain and fibre reinforced concrete. Cem. Concr. Compos..

[15] Kachkouch F.Z., Noberto C.C., de Albuquerque Lima Babadopulos L.F., Melo A.R.S., Machado A.M.L., Sebaibi N., Boukhelf F., El Mendili Y. (2022). Fatigue behavior of concrete: A literature review on the main relevant parameters. Constr. Build. Mater..

[16] Wu B., Jin H. (2019). Compressive fatigue behavior of compound concrete containing demolished concrete lumps. Constr. Build. Mater..

[17] Dong S., Wang Y., Ashour A., Han B., Ou J. (2021). Uniaxial compressive fatigue behavior of ultra-high performance concrete reinforced with super-fine stainless wires. Int. J. Fatigue.

[18] Becks H., Classen M. (2025). Influence of coarse aggregates on the fatigue behavior of high-strength concrete under mode II loading. Constr. Build. Mater..

[19] Ortega J.J., Ruiz G., Yu R.C., Afanador-García N., Tarifa M., Poveda E., Zhang X., Evangelista F. (2018). Number of tests and corresponding error in concrete fatigue. Int. J. Fatigue.

[20] Huang B.T., Li Q.H., Xu S.L., Zhou B.M. (2018). Tensile fatigue behavior of fiber-reinforced cementitious material with high ductility: Experimental study and novel P-S-N model. Constr. Build. Mater..

[21] Sohel K.M.A., Al-Jabri K., Zhang M.H., Liew J.Y.R. (2018). Flexural fatigue behavior of ultra-lightweight cement composite and high strength lightweight aggregate concrete. Constr. Build. Mater..

[22] Liu M., Lu J., Ming P., Yin Y. (2021). Study of fracture properties and post-peak softening process of rubber concrete based on acoustic emission. Constr. Build. Mater..

[23] Keerthana K., Kishen J.M.C. (2020). Micromechanics of fracture and failure in concrete under monotonic and fatigue loadings. Mech. Mater..

[24] De Smedt M., Vrijdaghs R., Van Steen C., Verstrynge E., Vandewalle L. (2020). Damage analysis in steel fibre reinforced concrete under monotonic and cyclic bending by means of acoustic emission monitoring. Cem. Concr. Compos..

[25] Radhika V., Kishen J.C. (2024). A comparative study of crack growth mechanisms in concrete through acoustic emission analysis: Monotonic versus fatigue loading. Constr. Build. Mater..

[26] Vicente M.A., Mínguez J., González D.C. (2019). Computed tomography scanning of the internal microstructure, crack mechanisms, and structural behavior of fiber-reinforced concrete under static and cyclic bending tests. Int. J. Fatigue.

[27] Fataar H., Combrinck R., Boshoff W.P. (2022). An Experimental Study on the Flexural Fatigue Behaviour of Pre-cracked Steel Fibre Reinforced Concrete. Fibre Reinforced Concrete: Improvements and Innovations II.

[28] Skarżyński Ł., Marzec I., Tejchman J. (2019). Fracture evolution in concrete compressive fatigue experiments based on X-ray micro-CT images. Int. J. Fatigue.

[29] Yang Z., Li H., Wen J., Huang F., Wang Z., Yi Z., Xie Y., Dong H. (2023). The microstructure evolution of ballastless track high-strength concrete exposed to compressive and flexural fatigue loads. Int. J. Fatigue.

[30] Fan Z., Sun Y. (2019). Detecting and evaluation of fatigue damage in concrete with industrial computed tomography technology. Constr. Build. Mater..

[31] Li Z., Shen A., Long H., Guo Y., He T. (2021). Dynamic deterioration of strength, durability, and microstructure of pavement concrete under fatigue load. Constr. Build. Mater..

[32] Gan Y., Breugel K., Schlangen E., Šavija B. Experimental And Numerical Study Of Fatigue Damage In Hardened Cement Paste At The Microscale. Proceedings of the 11th International Conference on Fracture Mechanics of Concrete and Concrete Structures.

[33] Li Q., Huang B., Xu S., Zhou B., Yu R.C. (2016). Compressive fatigue damage and failure mechanism of fiber reinforced cementitious material with high ductility. Cem. Concr. Res..

[34] Yang K., Zhuang S., Wang Y., Li J., Zhou S., Ren J. (2025). Crack Propagation of Ceramsite Lightweight Concrete Under Four-Point Bending Fatigue Conditions. Materials.

[35] Zhang B. (1998). Relationship Between Pore Structure and Mechanical Properties of Ordinary Concrete Under Bending Fatigue. Cem. Concr. Res..

[36] Qiu J., Yang E.H. (2017). Micromechanics-based investigation of fatigue deterioration of engineered cementitious composite (ECC). Cem. Concr. Res..

[37] Chen H., Song J., Li D. (2025). Fatigue Crack Propagation Properties of Ordinary Plain Concrete Under Three-Point Loading. Materials.

[38] Oneschkow N., Timmermann T. (2022). Influence of the composition of high-strength concrete and mortar on the compressive fatigue behaviour. Mater. Struct..

[39] Schäfer N., Breitenbücher R. Microcracking of high-performance concrete under cyclic loading and the influence of the aggregate. Proceedings of the 13th Fib International PhD Symposium in Civil Engineering.

[40] Oneschkow N., Scheiden T., Hüpgen M., Rozanski C., Haist M. (2021). Fatigue-Induced Damage in High-Strength Concrete Microstructure. Materials.

[41] Blasón S., Poveda E., Ruiz G., Cifuentes H., Fernández Canteli A. (2019). Twofold normalization of the cyclic creep curve of plain and steel-fiber reinforced concrete and its application to predict fatigue failure. Int. J. Fatigue.

[42] Blasón S., Fernández Canteli A., Poveda E., Ruiz G., Yu R.C., Castillo E. (2022). Damage evolution and probabilistic strain-lifetime assessment of plain and fiber-reinforced concrete under compressive fatigue loading: Dual and integral phenomenological model. Int. J. Fatigue.

[43] Song Y., Zhao D., Qin L. (2002). Sample size analyses for tests of concrete strength and fatigue life. J. Dalian Univ. Technol..

[44] Tepfers R., Kutti T. (1979). Fatigue Strength of Plain, Ordinary, and Lightweight Concrete. ACI J. Proc..

[45] Cachim P.B., Figueiras J.A., Pereira P.A.A. (2002). Fatigue behavior of fiber-reinforced concrete in compression. Cem. Concr. Compos..

[46] Li L., Xu L., Huang L., Xu F., Huang Y., Cui K., Zeng Y., Chi Y. (2022). Compressive fatigue behaviors of ultra-high performance concrete containing coarse aggregate. Cem. Concr. Compos..

[47] Mun J.S., Yang K.H., Kim S. (2016). Tests on the Compressive Fatigue Performance of Various Concretes. J. Mater. Civ. Eng..

[48] Lemaitre J. (1985). A continuous damage mechanics model for ductile fracture. J. Eng. Mater. Technol..

